# Global prevalence of nitrofurantoin-resistant uropathogenic *Escherichia coli* (UPEC) in humans: a systematic review and meta-analysis

**DOI:** 10.1093/jac/dkaf305

**Published:** 2025-08-20

**Authors:** Christopher Larkin, Sabeel P Valappil, Navaneethan Palanisamy

**Affiliations:** Chester Medical School, University of Chester, Parkgate Road, Chester CH1 4BJ, UK; Mast Group Limited, Mast House, Derby Road, Bootle L20 1EA, UK; Chester Medical School, University of Chester, Parkgate Road, Chester CH1 4BJ, UK; Chester Medical School, University of Chester, Parkgate Road, Chester CH1 4BJ, UK

## Abstract

**Background:**

The global rise in antimicrobial resistance (AMR) is a significant health concern. Nitrofurantoin is used as a first-line antibiotic against many uropathogenic bacterial pathogens, including uropathogenic *Escherichia coli* (UPEC), and an analysis is required to assess the current global prevalence of nitrofurantoin-resistant UPEC.

**Methods:**

Following the Preferred Reporting Items for Systematic Reviews and Meta-Analyses (PRISMA) guidelines, a literature search using PubMed and Google Scholar was performed to find studies reporting nitrofurantoin-resistant UPEC in humans. Studies were included/excluded based on predefined criteria and focused only on isolates collected from the urinary tract. The quality of the studies was assessed using the Joanna Briggs Institute’s (JBI’s) Checklist for Prevalence Studies. Statistical analysis was performed using Metafor and Meta (R packages) to estimate the pooled prevalence, assess publication bias and perform heterogeneity analysis.

**Results:**

Sixty-three studies comprising 774 499 UPEC isolates collected between 1996 and 2024 were analysed and demonstrated a global pooled prevalence of nitrofurantoin-resistant UPEC isolates to be 6.9% (95% CI: 4.8%–9.7%). Continent-wise subgroup analysis showed Europe to have the lowest prevalence, while Asia has the highest prevalence. Decade-wise subgroup analysis showed the global prevalence increased from 2.8% (1996–04) to 8.2% (2005–14) and then decreased to 7.6% in the last decade (2015–24). Substantial heterogeneity was seen among the studies examined, as well as statistically significant publication bias.

**Conclusions:**

The findings show considerable global prevalence of nitrofurantoin-resistant UPEC isolates, with the prevalence being higher in low- and middle-income countries (LMICs). Sufficient education should be provided where possible, and antimicrobial stewardship should be intensified to slow the rate of AMR increase worldwide.

## Introduction

Urinary tract infections (UTIs) are among the most common infections in humans worldwide, posing a constant threat to human well-being, as well as the economic resources of society.^1^ UTIs manifest themselves in an extensive range of infections, which are broadly classified as uncomplicated UTIs (lower risk) and complicated UTIs (higher risk).^2^ Typically, UTIs are caused by colonization of the urethra by one or multiple bacterial species, but can also occur via haematogenous or lymphatic distribution of infection into the urinary tract.^1^

Uropathogenic *Escherichia coli* (UPEC) is the leading cause of UTIs globally,^3^ and can persistently grow within the urinary tract, making it a constant threat of infection.^4^ Persistent growth of UPEC is speculated to be due to their ability to remain in a dormant state within late endosomal compartments of cells in the urinary system, as shown in mice, which may enable them to evade the host immune response and pave the way for recurrent infections.^4^ A study has also demonstrated low abundance of bacterial cells in bladder biopsy samples that are not within cells but more predominantly in the intracellular spaces/intratissues.^5^ Once infection is established in the urinary tract (urethra), UPEC can spread to the bladder (cystitis), the kidneys (pyelonephritis) and eventually the bloodstream (urosepsis) if left untreated.^3^

Antimicrobial resistance (AMR) is one of the greatest threats to human health that we currently face, with acquired resistance to antimicrobials being directly responsible for 250 000 deaths in Africa and 1.27 million deaths worldwide in 2019.^6,7^ In bacteria, antibiotic resistance typically occurs due to genetic resistance factors, which may be intrinsic to the species, acquired via chromosomal gene mutation, via mobile genetic elements, or from adaptation to a selective pressure within an environment.^8^ Higher rates of AMR are observed in low- and middle-income countries (LMICs), and this is commonly due to a lack of awareness or understanding of the problem.^9^

Nitrofurantoin is a highly efficacious, orally taken antimicrobial that is commonly used to treat uncomplicated UTIs, including those caused by UPEC.^10^ This drug belongs to the nitrofuran group—a group of compounds that contain a nitro group (−NO_2_) on a furan ring.^11^ Nitrofurans are understood to be chemically activated by a reduction mechanism of the nitro group, making it highly reactive.^12,13^ Activation of nitrofurans and reduction of the nitro group occur due to nitroreductase activity,^12,13^ which can be seen in bacterial species that have functional *nfsA*, *nfsB* and *ribE* genes. Although the modes of action of nitrofurans are not yet fully understood,^14^ it is understood that resistance to nitrofurantoin occurs when the action of the above-listed genes has been impaired.^15,16^ Further, it has been identified that nitrofurantoin resistance can also be acquired via horizontal gene transfer (HGT). OqxAB, a novel multidrug efflux pump encoded on a conjugative plasmid, has been linked with nitrofurantoin resistance in *E. coli*.^17^ Also, mutations T55A, A273P and R277C in the CTX-M-14 enzyme, a type of extended-spectrum beta-lactamase (ESBL) enzyme, have been identified to confer resistance towards nitrofurantoin in *E. coli*.^18^

AMR is a constantly escalating global health threat, and unnecessary prescription of antibiotics for UTI treatments is a major contributing factor to AMR. This study aims to evaluate the global prevalence of nitrofurantoin-resistant UPEC isolates. Further, this study also aims to assess their prevalence in different continents and their prevalence dynamics in the last three decades. Our study will highlight whether there has been a significant increase/decrease in the prevalence of nitrofurantoin resistance in UPEC and also provide insight into a locus (e.g. continent/countries) in which such a problem may lie, thereby paving the way for intensified control measures/strategies to prevent further spread of these bacteria.

## Methods

The Preferred Reporting Items for Systematic Reviews and Meta-Analyses (PRISMA) 2020 Guidelines and PRISMA 2020 Checklist were followed to perform this systematic review and meta-analysis.^19^

### Literature search

PubMed and Google Scholar were used to perform a widespread literature search to find a complete scope of relevant published articles. The inputs (search strings) used for the literature search are shown in Table S1 (available as Supplementary data at *JAC* Online). In the case of Google Scholar, only the first 100 articles were considered for screening. The retrieved articles were manually scrutinized for any duplicates, all of which were removed when found. The searches were performed first on 20 April 2024 and then recently on 17 May 2025.

The titles and abstracts of all articles retrieved from the above search were assessed using the predefined inclusion and exclusion criteria that are given below. Studies containing patients of all ages and biological sexes were considered for analysis. Each article that met these criteria was considered for quality assessment.

### Inclusion and exclusion criteria

Inclusion and exclusion criteria were specified prior to the review of any articles. Studies were considered suitable for analysis if they were as follows:

- Primary research article
- Published in English
- Urinary samples were retrieved from humans
- Included at least one isolate of human UPEC
- Prevalence of UPEC nitrofurantoin resistance was reported

Studies were deemed unfit for analysis if:

- No English publication or translation was available
- Not a primary research article (e.g. systematic review and meta-analysis)
- Isolates were from non-human sources (e.g. animals and environmental)
- Isolates were not sourced from the urinary tract, or isolates from the urinary tract were not reported separately to isolates obtained from other sources (e.g. blood and stool)
- Isolates were pre-selected for resistance mechanisms (e.g. biofilm-forming *E. coli* and ESBL-producing *E. coli*)
- Nitrofurantoin resistance for *E. coli* was not reported
- Full article text was inaccessible or unavailable
- Unclear/unfit methods for analysing AMR were used
- The patient population was selected for underlying health conditions (e.g. diabetes and HIV)

The inclusion/exclusion criteria were reviewed by the senior author (N.P.). The review of whether each study was fit for analysis was performed by the authors independently. No automation tools were used during the review process. N.P. also acted as the referee to solve any discrepancies among the authors.

### Quality assessment

Quality assessment of the studies was performed using the Joanna Briggs Institute’s (JBI’s) ‘Checklist for Prevalence Studies’.^20^ Any paper that scored under 6 on this nine-question checklist was deemed unfit for analysis and was excluded. The questions are as follows:

1. Was the sample frame appropriate to the target population?
2. Were participants sampled appropriately?
3. Was an adequate sample size used?
4. Were the study participants and the setting described with enough detail?
5. Was the data analysis conducted with sufficient coverage of the identified sample?
6. Were valid methods used for identification?
7. Was measurement performed in a standard and reliable way for all participants?
8. Was an appropriate statistical analysis performed?
9. Was the response rate adequate?

To define an appropriate sample size for question 3, the Scalex Single Proportion (SP) calculator was used.^21^ A 95.0% confidence level, 10.0% expected prevalence, 0.0% expected loss and 5.0% precision were used as inputs. This provided an expected sample size of 139 clinical UPEC isolates, which was then used as the breakpoint for question 3.

Statistical analysis was considered adequate for question 8, so long as the percentage susceptibility or resistance to nitrofurantoin was presented in a clear manner. No further analysis was required by each study to be considered appropriate for this systematic review and meta-analysis.

Question 9 was deemed inappropriate for this study, and therefore, ‘Not applicable’ was answered for each study.

### Data collection

For a concise compilation of relevant information for this study, a Microsoft Excel database containing the following information from each article was created:

- Author’s name(s)
- Year of publication
- Study period
- Country of study
- Continent of study
- Patient population
- Number of UPEC isolates
- Antimicrobial susceptibility testing (AST) method used
- Nitrofurantoin concentration (μg)
- Percentage UPEC nitrofurantoin resistance (%)
- Number of nitrofurantoin-resistant UPEC isolates

In the event that the study period was reported year-by-year, the percentage of nitrofurantoin resistance was recorded as a mean value for the study period.

### Statistical analysis

Statistical analysis was carried out using Metafor and Meta packages on RStudio version 4.5.0. For data entry, the one-group dichotomous model was used, and the random effects model was used for analysis. *Q*- and *I*^2^-statistics were used to assess the heterogeneity of the data. Continental and decadal subgroup analysis was also performed by grouping the data accordingly. Meta-regression was used to determine statistically significant differences for each subgroup compared to the overall pooled prevalence, where *P* < 0.05 would define statistical significance. Multi-continental studies were excluded from the continental subgroup analysis, and studies not specifying a study period were excluded from the decadal subgroup analysis. Decadal subgroups were identified as 1996–04, 2005–14 and 2015–24. If a period of study overlapped with these subgroups, they were assigned to both groups. Stepwise omission sensitivity analysis was performed to see the impact of particular studies on the pooled prevalence by sequentially removing each study from the dataset. Publication bias was assessed using a funnel plot. Duval and Tweedie’s trim and fill method was followed to insert imputed studies and highlight the effect size of publication bias.^22^ This effect size was quantified using the Begg and Mazumdar rank correlation test.^23^ A *P* value of <0.05 was used to determine statistical significance.

## Results

### Literature search

**Figure 1.**
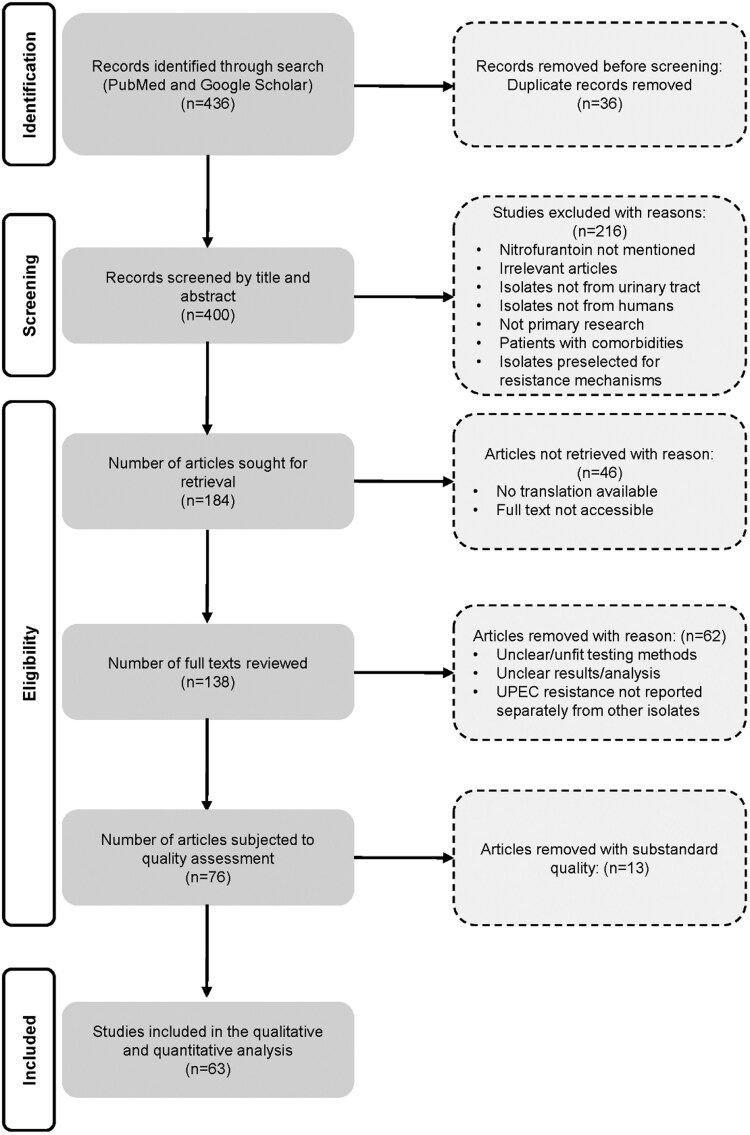
A PRISMA flow diagram showing the results of the literature searching and screening processes. UPEC, uropathogenic *Escherichia coli*.

The initial literature search of PubMed and Google Scholar resulted in a total of 436 articles, 36 of which were duplicates. After reviewing titles/abstracts, 216 articles were omitted for the reasons detailed in Figure 1. Additionally, 46 articles could not be obtained due to online unavailability or no translation being available. Upon reviewing the full texts, 62 articles were omitted due to unclear/unfit testing methods/results/analysis and UPEC resistance not reported separately from other isolates. The JBI quality assessment resulted in 13 articles being removed from further analysis, leaving 63 articles for the final analysis^24–86^. The quality assessment scores of the articles can be seen in Table S2.

### Study characteristics

**Figure 2.**
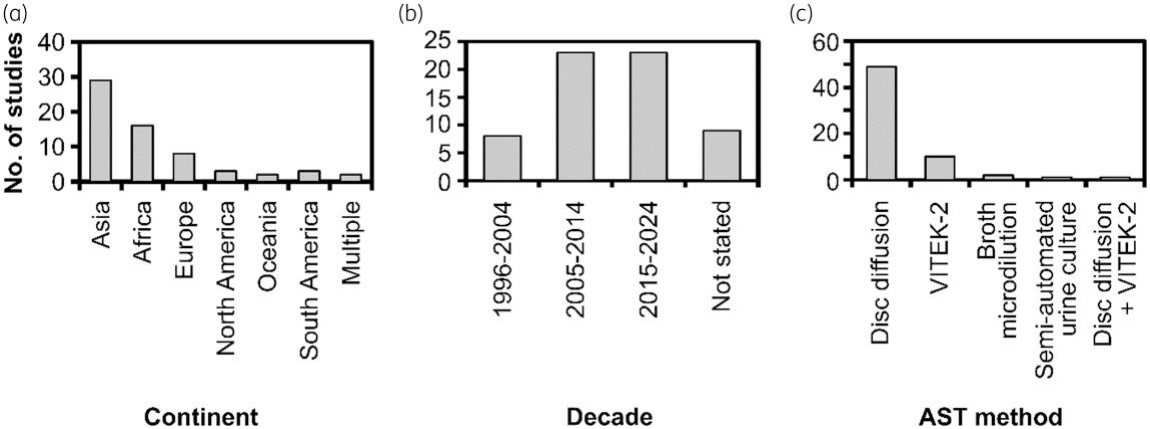
Overview of the included studies. Classification of studies based on (a) continent where the study was conducted, (b) decade during which the study was conducted and (c) antibiotic susceptibility test (AST) method used for screening the nitrofurantoin-resistant UPEC isolates.

A comprehensive table containing the extracted data from the articles can be seen in Table S3. From the 63 selected articles, a total of 774 499 UPEC isolates were analysed for nitrofurantoin resistance. A majority of these UPEC isolates were from a study conducted by Ong *et al.*^74^ in the UK, which analysed 712 004 UPEC isolates. Nearly a half and a quarter of these studies were conducted in Asian and African countries, respectively (Figure 2a). Two studies were conducted on multiple continents.^46,77^ Iran was the most frequently represented country, with data sourced from 11 studies,^29,32,40,43,44,54,56,71,79,81,85^ and conducted across multiple regions in the country. One of the 63 studies was stated to be performed outside of a clinical setting.^83^ Neuzillet *et al.*^70^ presented only the French results rather than the full ARESC study, which covered nine European countries and Brazil. Full ARESC results can be found in the study published by Schito *et al*.^87^ Schito *et al.*’s study was not included in our analysis as we were not able to find this study using our search strategy. The period covered by the selected studies spanned from 1996 to 2024. When split between decades, most of these studies were conducted in the last two decades (i.e. 2005–24: 23 studies in each decade) (Figure 2b). When classified based on the AST method used, most of these studies used the Kirby–Bauer disc diffusion method, followed by the VITEK-2 system (Figure 2c). Two studies used the broth microdilution method,^46,57^ and one study utilized a semi-automated urine culture system.^74^

### Global prevalence of nitrofurantoin-resistant UPEC

**Figure 3.**
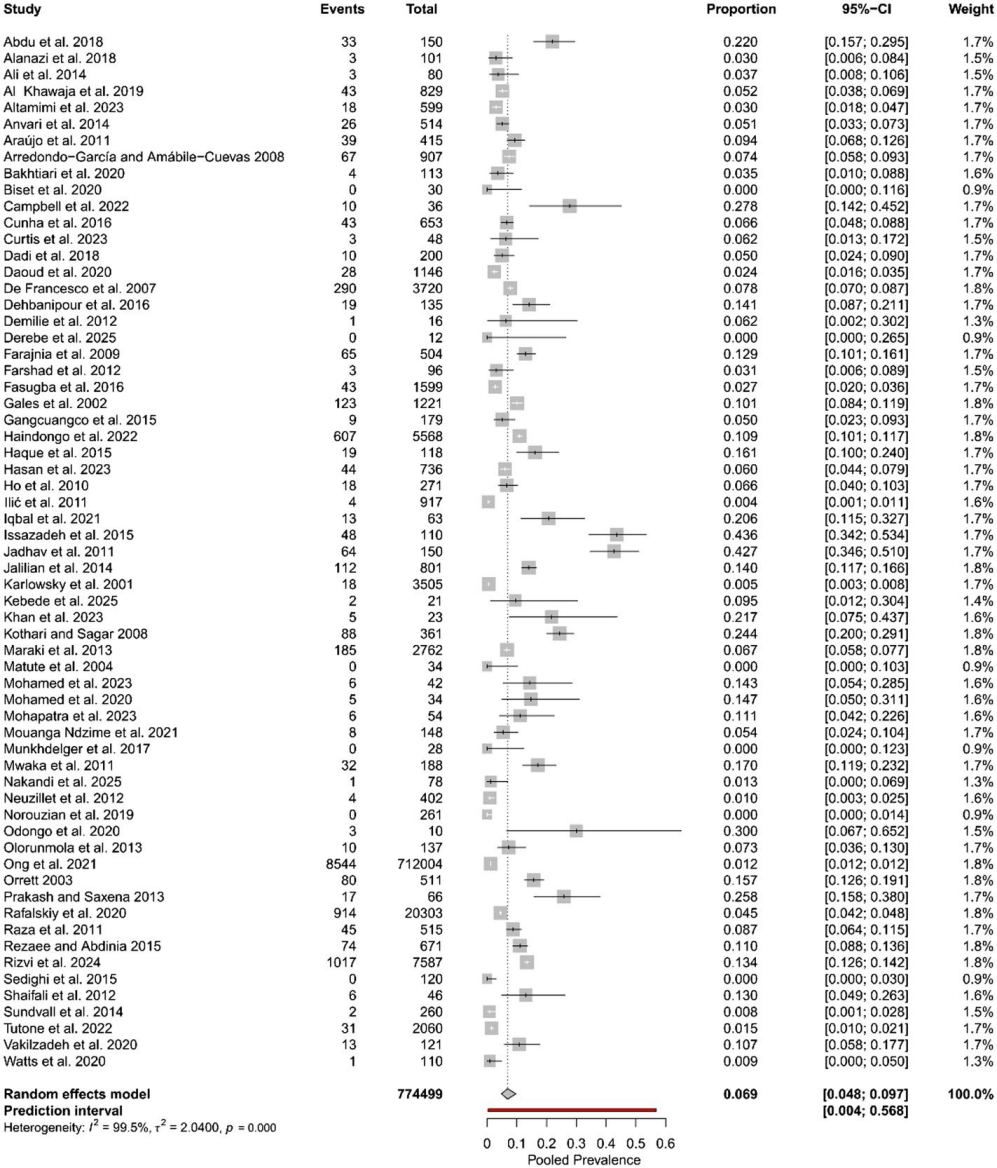
A forest plot showing the estimated global pooled prevalence of nitrofurantoin-resistant UPEC using data from 63 studies. The random effects model was used. CI, confidence interval. Analysis was done using Metafor and Meta packages on RStudio version 4.5.0. Proportion, events/total; percentage, proportion × 100; weight, influence of each study on the overall pooled result.

**Figure 4.**
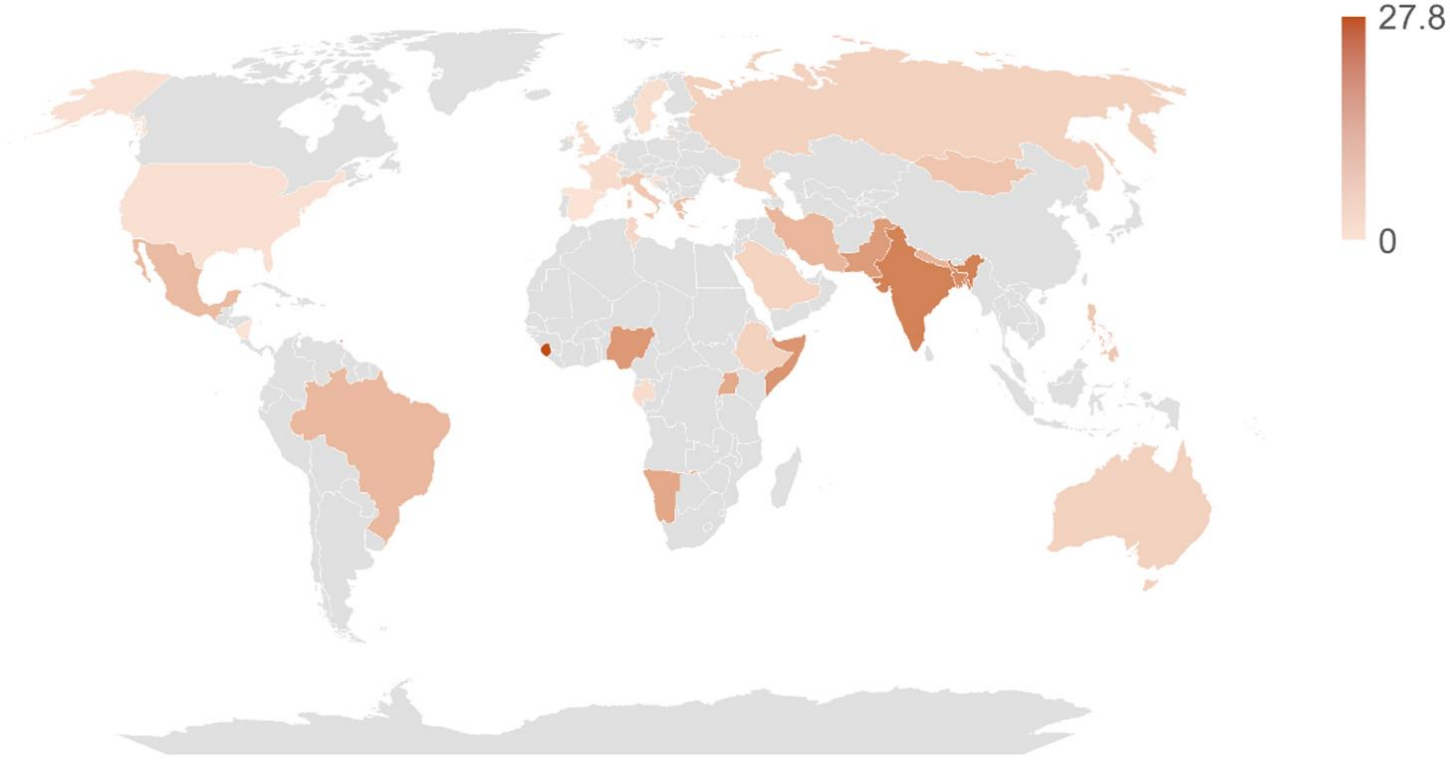
A global map illustrating the prevalence of nitrofurantoin-resistant UPEC isolates across various countries. Values given in %. Grey, studies not available from these countries, or not identified using our search strategy, or not included in our study due to not meeting the inclusion criteria. Data from Gales *et al.* (conducted in Latin American countries and Mexico) not included in this analysis/map due to a lack of country-wise information regarding nitrofurantoin-resistant UPEC isolates.

From the 63 selected studies, the estimated global pooled prevalence of nitrofurantoin-resistant UPEC was 6.9% (95% CI: 4.8%–9.7%) (Figure 3). The prediction interval was from 0.4% to 56.8%. The *Q*-value was 13 257.47 with 62 degrees of freedom, and the *I*^2^-value was 99.5%, demonstrating statistically significant heterogeneity with a *P* value of 0.000 (Figure 3). A global map illustrating the prevalence of nitrofurantoin-resistant UPEC isolates across various countries is shown in Figure 4. Country-wise, the prevalence of nitrofurantoin-resistant UPEC isolates was the highest in Sierra Leone (27.8%), followed by India with 18.3%, Bangladesh with 16.1%, Trinidad with 15.7%, Somalia with 14.5%, Nigeria with 13.9%, Pakistan with 13.3%, Namibia with 10.9% and Uganda with 10.7%. It should be noted that data from Sierra Leone, Bangladesh, Trinidad and Namibia were based on a single study each. The rest of the countries had <9% prevalence (Table S4). In the EU, Greece had the highest prevalence with 6.7% (based on a study by Maraki *et al.*) followed by Italy with 4.9%. In the UK, the prevalence was around 1.1%.

### Subgroup analysis

Continent-wise subgroup analysis revealed that Europe has the lowest prevalence of nitrofurantoin-resistant UPEC isolates and Asia has the highest, at 1.7% and 9.6%, respectively (Table 1). Statistically, the prevalence of nitrofurantoin-resistant UPEC isolates in Europe was significantly lower compared to the global prevalence (*P* = 0.000), while their prevalence was significantly higher in Asia compared to the global prevalence (*P* = 0.035). Their prevalence was also high in Africa (9.3%), and similar to Asia, but was not statistically significant when compared to the global prevalence (*P* = 0.166) (Table 1). For this subgroup analysis, the studies by Orrett in Trinidad and Matute *et al.* in Nicaragua were brought under North America and South America, respectively.^62,75^ When performing the decade-wise subgroup analysis, the period 1996–04 was estimated to have a prevalence of 2.8% (Table 1). In the subsequent decade (i.e. 2005–14), the prevalence increased by 5.4% points to 8.2% and then decreased by 0.6% points to 7.6% in the last decade (i.e. 2015–24) (Table 1).

**Table 1.** Subgroup analysis of the included studies

Subgroup	Number of Studies	UPEC isolates	Nitrofurantoin-resistant UPEC isolates	Pooled prevalence	CI lower	CI upper	*P* value	*Q*-value	*I* ^2^-value
Continent									
Africa	16^24,33,34,37,38,41,42,48,58,63,64,67–69,72,73^	7696	748	0.093	0.055	0.152	0.166	191.55	92%
Asia	29^25–29,32,40,43,44,47,49–51,53–56,59,60,65,66,71,76,78–82,85^	15 372	1790	0.096	0.068	0.135	0.035	611.37	95%
Europe	8^39,52,61,70,74,83,84,86^	722 235	9061	0.017	0.008	0.036	0.000	705.45	99%
North America	3^31,57,75^	4923	165	0.053	0.021	0.127	0.714	401.73	99%
Oceania	2^36,45^	1647	46	0.039	0.009	0.147	0.07	-	-
South America	3^30,35,62^	1102	82	0.063	0.019	0.191	0.533	7.94	74%
Decade									
1996–04	8^39,46,52,57,62,70,73,75^	10 707	531	0.028	0.013	0.058	0.017	584.44	99%
2005–14	23^25,26,29,30,35,38,40,41,45,47,49,51,54–56,60,61,66,76,78,79,81,83^	10 584	880	0.082	0.053	0.124	0.396	1701.57	99%
2015–24	23^27,28,33,34,36,42,48,50,53,58,59,63,64,68,69,71,72,74,77,80,84–86^	751 909	11 340	0.076	0.047	0.120	0.653	1016.65	98%

A hyphen (-) is in place where a value could not be obtained.

UPEC, uropathogenic *Escherichia coli*; CI, confidence interval.

Heterogeneity analysis was conducted to identify variability in the results of individual studies included in the analysis. Cochran’s *Q* test and *I*^2*-*^statistic are used for this purpose. While the *Q* test checks for the presence of heterogeneity, the *I*^²^ estimates the proportion of variance due to heterogeneity. A low heterogeneity (i.e. *I*^2^ closer to 0%; low *Q* scores) indicates reliability and generalizability of the results and vice versa if higher (*I*^2^ > 50%; higher *Q* scores). A high heterogeneity was observed in all continental and decadal subgroups, excluding South America, which demonstrated homogeneous effect sizes throughout the three studies within the subgroup (*Q* = 7.94) (Table 1).^30,35,62^ However, the *I*^2^-value for the South American subgroup was 74%, implying that only 74% of the observed variance reflects variance in true effects. The lack of confidence in true variance is likely caused by the low number of isolates sampled in the study conducted by Matute *et al.*^62^ (*n* = 34).

### Sensitivity analysis

Sensitivity analysis of 62 out of the 63 studies showed that the global pooled prevalence remained consistent, ranging between 6.6% and 7.2% (Table S5). Heterogeneity was unaffected, with the *I*^2^-value remaining 100% for each iteration of removal (Table S5). The study that produced a noticeable difference in both pooled prevalence and the *Q*-statistic was by Ong *et al*.^74^ The removal of this study from the analysis resulted in a pooled prevalence of 7.7% and a *Q*-statistic of 1815.403, compared to the average *Q*-statistic of 12 917.36 found in this sensitivity analysis (Table S5). A *t*-test was performed on the pooled prevalence with Ong *et al.*^74^ removed compared to the full cohort, and no statistical significance was observed (*P* = 0.6198).

### Publication bias

Publication bias analysis identifies if there is any overestimation of effect size due to the tendency for studies with positive results (i.e. studies with statistically significant results) to be more likely to be published than studies with negative results. A funnel plot was used to study the publication bias. Thirty-nine of the 63 included studies were found to lie outside of the 95% CI of the overall effect size (Figure S1a).^24,28,30,33,34,38–40,42,43,45,46,48,49,52–57,59,60,62,67–72,74–77,79–81,83,84,86^ Using Duval and Tweedie’s trim and fill method,^22^ an additional 41 studies were imputed (Figure S1b). Kendall’s Tau-b value calculated from the Begg and Mazumdar rank correlation test was 0.536, with a one-tailed *P* value of 0.000. This shows that statistically significant publication bias was present in this analysis.

## Discussion

Bacterial pathogens cause a range of diseases in humans. In many cases, without any interventions, such as antibiotics, the prognosis is very poor. The number of effective antibiotics in our arsenal for combating bacteria is diminishing at an accelerating rate due to the rise in resistant bacterial strains. UTIs are common human infections, primarily caused by bacteria, particularly UPEC, and require antibiotic treatment. Nitrofurantoin is utilized as a first-line therapy for uncomplicated UTIs and as prophylaxis for individuals prone to recurrent infections.^88^ Due to their extensive use, nitrofurantoin-resistant bacteria have been identified in clinical samples worldwide, and this is an irreversible evolution that is linked with extensive resistance.^89^ Documenting their prevalence region-/country-wise over the period will help us in understanding their dynamics and assist in developing effective strategies to mitigate their rapid transmission within the human population. Therefore, the present systematic review and meta-analysis aimed to assess the global prevalence of nitrofurantoin-resistant UPEC isolates in humans. The analysis included studies from all major continents, excluding Antarctica, and examined UPEC isolates dating back to 1996. This comprehensive approach has facilitated the evaluation of differences in prevalence levels across continental and decadal subgroups, highlighting potential trends in the rising levels of nitrofurantoin resistance by both time and location. To our knowledge, this is the first systematic review and meta-analysis in this endeavour.

The global pooled prevalence of nitrofurantoin-resistant UPEC was estimated at 6.9% (Figure 3). Its prevalence in Europe was lower (estimated at 1.7%) compared to the global prevalence and higher in Asia (9.6%). When comparing the differences in prevalence between Europe and Asia with the global prevalence, they are statistically significant (*P* = 0.000 and *P* = 0.035, respectively) (Table 1). Prevalence in Africa (9.3%), although not statistically significant compared to the global prevalence, remains high and similar to the prevalence in Asia (Table 1). This highlights the elevated rates of nitrofurantoin resistance in UPEC isolates in LMICs (Figure 4 and Table S4). A study conducted by Bryce *et al.*,^90^ who assessed the global prevalence of antibiotic resistance in paediatric UPEC infections, observed a pooled prevalence for nitrofurantoin resistance of 1.3% in countries within the Organisation for Economic Co-operation and Development (OECD). For countries outside of this organization, they observed a pooled prevalence of 17.0%.^90^ This study largely supports our findings, as the pooled prevalence of OECD countries is comparable to that observed in the European subgroup assessed in the present study. However, we found a 7.4% point lower prevalence in Asian countries, a 7.7% point lower prevalence in African countries and a 10.7% point lower prevalence in South American countries when compared to the pooled prevalence estimated for countries outside this organization. This difference is more likely due to study selection. It is unlikely that the overall prevalence of nitrofurantoin resistance would increase simply because the isolates assessed in the study conducted by Bryce *et al.*^90^ were exclusively taken from children. Similar to the present study, Bryce *et al.*^90^ analysed 58 articles. A limitation of their study was that publication bias was not reported. A funnel plot or quantitative publication bias analysis may have provided more insights into the reason for the disparity between our study and theirs.

A systematic review and meta-analysis conducted by Mortazavi-Tabatabaei *et al.*^91^ in 2019 focused exclusively on resistance in uropathogens across Iran and revealed that the pooled prevalence of nitrofurantoin-resistant UPEC in Iran was 18%. In comparison, the present study demonstrates a pooled prevalence of 8.2%, suggesting that the pooled prevalence in Iran is slightly less than that of the Asian continent (9.6%). Ninety studies were included in the analysis carried out by Mortazavi-Tabatabaei *et al.*,^91^ with strict inclusion/exclusion criteria not being applied, and no assessment of potential publication bias was incorporated. These limitations cast doubt on the predicted pooled prevalence of nitrofurantoin-resistant UPEC in Iran, and an updated analysis could be performed to obtain a more accurate estimation.

Similar to the meta-analysis performed by Mortazavi-Tabatabaei *et al.*, Tuem *et al.*^92^ conducted a meta-analysis on AMR in *E. coli* across Ethiopia. The pooled prevalence calculated for nitrofurantoin resistance in *E. coli* was 13.55%, which is notably higher than the Ethiopian and African pooled prevalences of 3.1% and 9.3%, respectively, calculated in the present study.^92^ However, it should be noted that Tuem *et al.*’s^92^ work was not limited to urinary pathogens. The three studies conducted in Ethiopia that are included in the present study reported resistances of 0.0%, 5.0%, and 6.3%, respectively.^33,37,41^ As the number of studies is limited, further studies should be performed to demonstrate the true prevalence of nitrofurantoin resistance in UPEC across Ethiopia.

As shown by the sensitivity analysis (Table S5), the largest impact on study heterogeneity came from the removal of the study conducted by Ong *et al.*^74^ in the UK (*Q* = 1815.403, *I*^2^ = 100%). This is likely because this study analysed a total of 712 004 isolates, which is over 10-fold higher than the number of UPEC isolates analysed over the other 62 studies combined (*n* = 67 940).^74^ The removal of this study increased the global pooled prevalence to 7.7%, compared to 6.9%, which again supports that LMICs have a higher prevalence of nitrofurantoin-resistant bacteria. In the UK, the low prevalence (1.1%) may be due to the limited use of nitrofurantoin until 2016 for treating uncomplicated acute UTI.^93^ It could also be argued that the inclusion of Ong *et al.*’s^74^ work within this meta-analysis is the reason for such a high level of publication bias (Figure S1), as the large population size of this study has skewed the event rate towards the left, resulting in a large number of the included studies hanging outside of the 95% CI. The 7.7% global pooled prevalence obtained from the exclusion of the study performed by Ong *et al.*^74^ is likely to be closer to the true pooled prevalence due to the lower prevalence-skewed bias that this study provides.

The present study shows a larger proportion of nitrofurantoin-resistant UPEC isolates within LMICs, especially India, Bangladesh, Pakistan, Nigeria, etc. This could be due to the cost of antibiotics/affordability, self-medication practice without prescription, lack of knowledge about AMR, etc. In India, a defined daily dose (DDD) of nitrofurantoin and co-trimoxazole (sulfamethoxazole + trimethoprim) in 2020 cost $0.023–$0.19 and $0.037–$0.04, respectively, which is comparably cheaper than other antibiotics and the cost of antibiotics in Europe.^94^ So, many patients can afford nitrofurantoin in India. In a study conducted in six LMICs on antibiotic access and use, the authors identified Bangladesh, along with Vietnam, as having the largest proportions of non-licensed antibiotic dispensing points.^95^ Drug stores were the most common point of contact for patients/sufferers when looking for antibiotics in these countries.^95^ The same study also identified that 45.7% of antibiotics were dispensed without a prescription in Bangladesh.^95^ These factors could contribute to the increased prevalence of nitrofurantoin-resistant UPEC isolates in these countries. To prolong the use of antibiotics in global medicine, further education should be provided where possible within these countries to slow the rate of AMR acquisition in bacterial species worldwide. Countries should continue to consider the introduction and encouragement of antimicrobial stewardship, following the lead of India and South Africa.^96,97^

A 2018 report by the European Centre for Disease Prevention and Control (ECDC) on the antimicrobial consumption in 28 European Union (EU) member states and two European Economic Area (EEA)/non-EU countries (Iceland and Norway) between 2013 and 2014 highlighted that 13 antibacterial agents, including nitrofurantoin, accounted for >50% of the consumption of antibacterials for systemic use in the community.^98^ The average consumption of nitrofuran derivatives in the UK in 2014 was estimated at 0.87 DDD per 1000 inhabitants per day, which was higher than the average estimated for the EU/EEA countries (0.72) in 2014.^98^ To our knowledge, this is the latest report we could access on the antimicrobial consumption in the community in the EU/EEA countries, and also, this report is not specific to nitrofurantoin but nitrofuran derivatives in general. Nevertheless, we attempted a correlation analysis and found no direct correlation between antibiotic consumption and prevalence of nitrofurantoin-resistant UPECs in these countries: in Greece, Italy, Belgium, France, Croatia, Sweden and Spain, the nitrofuran derivative consumption in 2014 was around 0.55, 0.22, 2.58, 0.18, 0.38, 0.78 and 0.13 DDD per 1000 inhabitants per day, respectively, and the prevalence of nitrofurantoin-resistant UPECs in these countries was 6.7%, 4.9%, 1.4%, 1%, 0.8, 0.4% and 0%, respectively. More studies on the prevalence of nitrofurantoin-resistant UPECs in different EU/EEA countries and up-to-date antimicrobial consumption data from these countries are needed to understand the true correlation (if any). A fairly recent prescription data, provided by the UK’s Office for Health Improvement and Disparities, highlighted a steady increase in nitrofurantoin prescription from 35.3% in 2016 to 73.1% in 2023 for England.^93^ Further studies are needed to understand whether increased nitrofurantoin usage in England in recent years has led to an increase in the prevalence of nitrofurantoin-resistant UPECs.

In recent years, an increase in MDR and XDR UPEC strains has been isolated.^99^ Antimicrobial combination therapy could increase the resistance barrier, thereby aiding in prolonging the usefulness of the antibiotics. They can also be used to treat these MDR and XDR isolates. A study conducted by Zhong *et al.*^100^ in 2020 utilized a *Galleria mellonella* (the greater wax moth or honeycomb moth) model and showed promising results concerning the combination of nitrofurantoin and amikacin as a tool against MDR UPEC. So, the usefulness of nitrofurantoin is still significant, and its usage should be carefully monitored.

To increase the longevity of nitrofurantoin usage in treating UPEC, it is recommended to identify the causative agent and test for resistance either phenotypically or genotypically before prescribing this antibiotic to the patients, as different uropathogenic bacteria have different resistance profiles to this antibiotic. In case of genotypic assessment, whole-genome or gene sequencing of clinical isolates has been proposed; however, it has been reported that the detection of nitrofurantoin-resistant isolates is not consistently achievable via this method.^14^ This is because several isolates have demonstrated a loss of function in the *nsfA* and *nsfB* genes, yet still exhibited susceptibility to nitrofurantoin.^14^ It is also recommended that physicians are regularly updated about the prevalence of nitrofurantoin-resistant strains circulating locally so that they can make informed decisions when prescribing this antibiotic to patients and also ensure that the urine concentration of this antibiotic in patients does not enter the selective window that drives mutation of *nfsA*.

The present study has some limitations. The number of studies included within each subgroup and the number of UPEC isolates in these studies varied widely and have limited the outcome of both continental/country-wise and decadal analyses. We relied on only PubMed and Google Scholar (the first 100 articles) in the present study, and we may have missed some articles (including grey literature). Further, we excluded 46 articles due to a lack of access at our institution and/or a lack of English translation. Furthermore, we did not include data from Schito *et al.*^87^ as we did not identify this study using our search strategy. It should be noted that in the study by Schito *et al.*, the authors identified the prevalence of nitrofurantoin-resistant UPEC isolates in Austria, Brazil, Germany, Hungary, Italy, Poland, Russia, Spain and the Netherlands to be 0%, 2.4%, 2.5%, 0%, 0%, 4.4%, 1.3%, 2.2% and 0%, respectively, among 2315 UPEC isolates. Including data from these missed articles might impact the estimated pooled prevalence.

## Conclusion

The results of the present study show that the global pooled prevalence of nitrofurantoin resistance in UPEC is 6.9%, although the true pooled prevalence could be closer to 7.7%. Over the decades, the prevalence has increased considerably (from 2.8% in 1996–04% to 7.6% in the last decade). The prevalence is quite high in Asia and Africa (i.e. in LMICs). In consideration of the future, antimicrobial stewardship and appropriate education should be provided worldwide, especially in continents/countries with comparatively higher prevalences, to reduce the rate of AMR growth and increase the longevity of nitrofurantoin.

## Supplementary Material

dkaf305_Supplementary_Data

## Supplementary data

Figure S1 and Tables S1–S5 are available as Supplementary data at *JAC* Online.
