# Supplementary material for: Global prevalence of nitrofurantoin-resistant uropathogenic Escherichia coli (UPEC) in humans: a systematic review and meta-analysis
Source: J Antimicrob Chemother. 2025 Aug 20;80(10):2609–21. doi: 10.1093/jac/dkaf305 (PMC12494140; doi:10.1093/jac/dkaf305)
Supplement: dkaf305_Supplementary_Data [file dkaf305_supplementary_data.docx]

**Supplementary Materials**

**Global Prevalence of Nitrofurantoin-Resistant Uropathogenic *Escherichia coli* (UPEC) in Humans: A Systematic Review and Meta-Analysis**

Christopher LARKIN^1,2^, Sabeel P. VALAPPIL^1^, Navaneethan PALANISAMY^1,^*

^1^Chester Medical School, University of Chester, Parkgate Road, Chester, CH1 4BJ, UK

^2^Mast Group Limited, Mast House, Derby Road, Bootle, L20 1EA, UK

*Corresponding author

Navaneethan Palanisamy ([n.palanisamy@chester.ac.uk](mailto:n.palanisamy@chester.ac.uk))

**Short running title:** Global Prevalence of Nitrofurantoin-Resistant Uropathogenic *Escherichia coli*

**
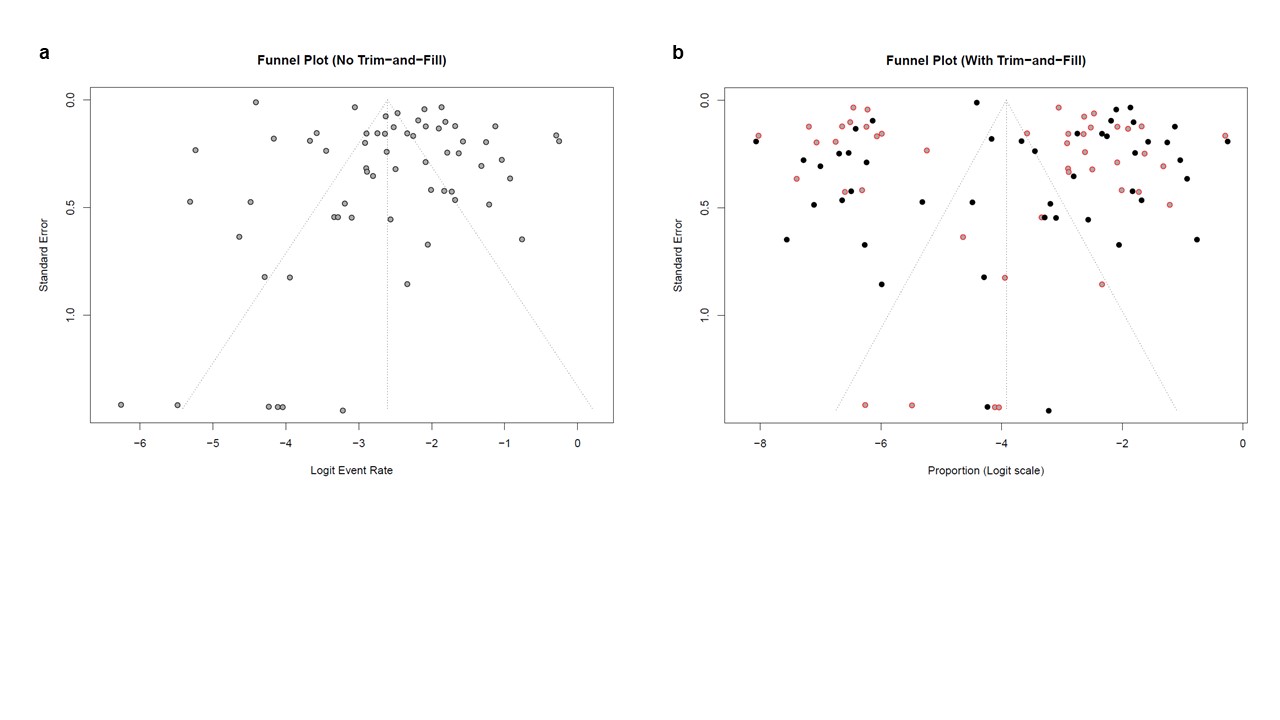
**

**Figure S1.** Funnel plot to study the publication bias. a) before applying the trim-and-fill method. b) after applying the trim-and-fill method (41 imputed studies shown in red).

**Table S1.** Queries used to search for relevant articles using MeSH terminology

| **Database** | **Full Query** |
| --- | --- |
| PubMed | ((Escherichia coli) OR (E. coli)) AND ((Uropathogenic) OR (UPEC)) AND (Nitrofurantoin) AND ((Antibiotic resistan*) OR (Antimicrobial resistan*)) AND ((Prevalence) OR (Epidemiology) OR (Rate) OR (Frequency)) |
| Google Scholar | Escherichia \| E. coli + Uropathogenic \| UPEC + Nitrofurantoin resistan* \| Antibiotic resistan* \| Antimicrobial resistan* + Prevalence \| Epidemiology \| Frequency \| Rate |

**Table S2.** Quality assessment of studies using the Joanna Briggs Institute’s (JBI’s) critical appraisal checklist for prevalence studies^1^

| **Study** | **Q1** | **Q2** | **Q3** | **Q4** | **Q5** | **Q6** | **Q7** | **Q8** | **Q9** | **Total Score** |
| --- | --- | --- | --- | --- | --- | --- | --- | --- | --- | --- |
| Abdu et al., 2018^2^ | Y | Y | Y | Y | Y | Y | Y | Y | N/A | 8 |
| Abduzaimovic et al., 2016^3^ | U | U | Y | N | Y | Y | Y | Y | N/A | 5 |
| Adib et al., 2014^4^ | Y | Y | N | Y | N | Y | Y | N | N/A | 5 |
| Alanazi et al., 2018^5^ | Y | Y | N | Y | N | Y | Y | Y | N/A | 6 |
| Ali et al., 2014^6^ | Y | Y | Y | Y | Y | Y | Y | Y | N/A | 8 |
| Al Khawaja et al., 2019^7^ | Y | Y | Y | Y | Y | Y | Y | Y | N/A | 8 |
| Altamimi et al., 2023^8^ | Y | Y | Y | Y | Y | Y | Y | Y | N/A | 8 |
| Anvari et al., 2014^9^ | Y | Y | Y | Y | Y | Y | Y | Y | N/A | 8 |
| Araújo et al., 2011^10^ | Y | Y | Y | Y | Y | Y | Y | Y | N/A | 8 |
| Arredondo-García and Amábile-Cuevas, 2008^11^ | Y | Y | Y | N | Y | Y | Y | Y | N/A | 7 |
| Bakhtiari et al., 2020^12^ | Y | Y | N | Y | N | Y | Y | Y | N/A | 6 |
| Bhola et al., 2020^13^ | U | U | Y | N | Y | Y | Y | Y | N/A | 5 |
| Biset et al., 2020^14^ | Y | Y | N | Y | N | Y | Y | Y | N/A | 6 |
| Campbell et al., 2022^15^ | Y | Y | N | Y | N | Y | Y | Y | N/A | 6 |
| Caracciolo et al., 2011^16^ | Y | Y | N | Y | N | Y | Y | N | N/A | 5 |
| Carmona-Cartaya et al., 2022^17^ | U | U | Y | N | Y | Y | Y | Y | N/A | 5 |
| Cunha et al., 2016^18^ | Y | U | Y | Y | Y | Y | Y | Y | N/A | 7 |
| Curtis et al., 2023^19^ | Y | Y | N | Y | N | Y | Y | Y | N/A | 6 |
| Dadi et al., 2018^20^ | Y | Y | Y | Y | Y | Y | Y | Y | N/A | 8 |
| Daoud et al., 2020^21^ | Y | Y | Y | Y | Y | Y | Y | Y | N/A | 8 |
| De Francesco et al., 2007^22^ | Y | U | Y | N | Y | Y | Y | Y | N/A | 6 |
| Dehbanipour et al., 2016^23^ | Y | Y | N | Y | N | Y | Y | Y | N/A | 6 |
| Demilie et al., 2012^24^ | Y | Y | N | Y | N | Y | Y | Y | N/A | 6 |
| Derebe et al., 2025^25^ | Y | Y | N | Y | N | Y | Y | Y | N/A | 6 |
| Farajnia et al., 2009^26^ | Y | Y | Y | Y | Y | Y | Y | Y | N/A | 8 |
| Farshad et al., 2012^27^ | Y | Y | N | Y | N | Y | Y | Y | N/A | 6 |
| Fasugba et al., 2016^28^ | Y | Y | Y | Y | Y | Y | Y | Y | N/A | 8 |
| Gales et al., 2002^29^ | Y | Y | Y | Y | Y | Y | Y | Y | N/A | 8 |
| Gangcuangco et al., 2015^30^ | Y | Y | Y | Y | Y | Y | Y | Y | N/A | 8 |
| Gatya Al-Mayahie et al., 2022^31^ | U | U | N | N | N | Y | Y | Y | N/A | 3 |
| Haghighatpanah and Mojtahedi, 2019^32^ | U | Y | N | N | N | Y | Y | Y | N/A | 4 |
| Haindongo et al., 2022^33^ | Y | Y | Y | Y | Y | Y | Y | Y | N/A | 8 |
| Haque et al., 2015^34^ | Y | Y | N | Y | N | Y | Y | Y | N/A | 6 |
| Hasan et al., 2023^35^ | U | Y | Y | N | Y | Y | Y | Y | N/A | 6 |
| Ho et al., 2010^36^ | Y | Y | Y | Y | Y | Y | Y | Y | N/A | 8 |
| Ilić et al., 2011^37^ | Y | Y | Y | Y | Y | Y | Y | Y | N/A | 8 |
| Iqbal et al., 2021^38^ | Y | Y | N | Y | N | Y | Y | Y | N/A | 6 |
| Issazadeh et al., 2015^39^ | Y | Y | N | Y | N | Y | Y | Y | N/A | 6 |
| Jadhav et al., 2011^40^ | Y | Y | Y | Y | Y | Y | Y | Y | N/A | 8 |
| Jalilian et al., 2014^41^ | U | Y | Y | N | Y | Y | Y | Y | N/A | 6 |
| Karlowsky et al., 2001^42^ | Y | Y | Y | Y | Y | Y | Y | Y | N/A | 8 |
| Kebede et al., 2025^43^ | Y | Y | N | Y | N | Y | Y | Y | N/A | 6 |
| Khan et al., 2023^44^ | Y | Y | N | Y | N | Y | Y | Y | N/A | 6 |
| Kothari and Sagar, 2008^45^ | Y | Y | Y | Y | Y | Y | Y | Y | N/A | 8 |
| Kumar et al., 2022^46^ | U | U | N | N | N | Y | Y | Y | N/A | 3 |
| Malik et al., 2021^47^ | Y | U | N | N | N | Y | Y | Y | N/A | 4 |
| Maraki et al., 2013^48^ | Y | Y | Y | Y | Y | Y | Y | Y | N/A | 8 |
| Matute et al., 2004^49^ | Y | Y | N | Y | N | Y | Y | Y | N/A | 6 |
| Mohamed et al., 2023^50^ | Y | Y | N | Y | N | Y | Y | Y | N/A | 6 |
| Mohamed et al., 2020^51^ | Y | Y | N | Y | N | Y | Y | Y | N/A | 6 |
| Mohapatra et al., 2023^52^ | Y | Y | N | Y | N | Y | Y | Y | N/A | 6 |
| Momtaz et al., 2013^53^ | Y | U | N | N | N | Y | Y | Y | N/A | 4 |
| Mouanga Ndzime et al., 2021^54^ | Y | Y | N | Y | N | Y | Y | Y | N/A | 6 |
| Munkhdelger et al., 2017^55^ | Y | U | Y | N | Y | Y | Y | Y | N/A | 6 |
| Mwaka et al., 2011^56^ | Y | Y | N | Y | N | Y | Y | Y | N/A | 6 |
| Nakandi et al., 2025^57^ | Y | Y | Y | N | Y | Y | Y | Y | N/A | 7 |
| Neuzillet et al., 2012^58^ | Y | Y | Y | Y | Y | Y | Y | Y | N/A | 8 |
| Nivetha et al., 2025^59^ | Y | Y | N | N | N | Y | Y | Y | N/A | 5 |
| Norouzian et al., 2019^60^ | Y | Y | Y | Y | Y | Y | Y | Y | N/A | 8 |
| Odongo et al., 2020^61^ | Y | Y | N | Y | N | Y | Y | Y | N/A | 6 |
| Olorunmola et al., 2013^62^ | Y | Y | N | Y | N | Y | Y | Y | N/A | 6 |
| Ong et al., 2021^63^ | Y | Y | Y | Y | Y | Y | Y | Y | N/A | 8 |
| Orrett, 2003^64^ | Y | Y | Y | Y | Y | Y | Y | Y | N/A | 8 |
| Prakash and Saxena, 2013^65^ | Y | Y | N | Y | N | Y | Y | Y | N/A | 6 |
| Rafalskiy et al., 2020^66^ | Y | Y | Y | Y | Y | Y | Y | Y | N/A | 8 |
| Raza et al., 2011^67^ | Y | Y | Y | Y | Y | Y | Y | Y | N/A | 8 |
| Rezaee and Abdinia, 2015^68^ | Y | Y | Y | Y | Y | Y | Y | Y | N/A | 8 |
| Rizvi et al., 2024^69^ | Y | Y | Y | N | Y | Y | Y | Y | N/A | 7 |
| Sedighi et al., 2015^70^ | Y | Y | N | Y | N | Y | Y | Y | N/A | 6 |
| Shaifali et al., 2012^71^ | Y | Y | N | Y | N | Y | Y | Y | N/A | 8 |
| Shehabi et al., 2004^72^ | U | U | Y | N | Y | Y | Y | Y | N/A | 5 |
| Sundvall et al., 2014^73^ | Y | Y | Y | Y | Y | Y | Y | Y | N/A | 8 |
| Tutone et al., 2022^74^ | Y | Y | Y | Y | Y | Y | Y | Y | N/A | 8 |
| Vakilzadeh et al., 2020^75^ | Y | Y | N | Y | N | Y | Y | Y | N/A | 6 |
| Watts et al., 2020^76^ | Y | Y | N | Y | N | Y | Y | Y | N/A | 6 |
| Yilmaz et al., 2016^77^ | U | U | Y | N | Y | Y | Y | Y | N/A | 5 |

Y – Yes. N – No. U – Unclear. N/A – Not Applicable.

Q1. Was the sample frame appropriate to the target population?

Q2. Were participants sampled appropriately?

Q3. Was an adequate sample size used?

Q4. Were study participants and the setting described with enough detail?

Q5. Was the data analysis conducted with sufficient coverage of the identified sample?

Q6. Were valid methods used for identification?

Q7. Was measurement performed in a standard, and reliable way for all participants?

Q8. Was appropriate statistical analysis performed?

Q9. Was the response rate adequate?

| **Table S3.** Characteristics of included studies | | | | | | | | | |  |
| --- | --- | --- | --- | --- | --- | --- | --- | --- | --- | --- |
| **Author's Name** | **Year of Publication** | **Study Period** | **Country/Territory of Study** | **Continent of Study** | **Patient Population** | **Number of UPEC Isolates Tested** | **AST Method** | **Nitrofurantoin Concentration (μg)** | **Nitrofurantoin-Resistant UPEC Isolates** | **Percentage UPEC Nitrofurantoin Resistance (%)** |
| Abdu et al.^2^ | 2018 | Not stated | Nigeria | Africa | Ages 15 to >=50 | 150 | Disc diffusion | 50 | 33 | 22.0 |
| Alanazi et al.^5^ | 2018 | 2008 | Saudi Arabia | Asia | All ages | 101 | VITEK-2 | Not stated | 3 | 3.0 |
| Ali et al.^6^ | 2014 | 2012-2013 | Pakistan | Asia | All ages | 80 | Disc diffusion | 300 | 3 | 3.8 |
| Al Khawaja et al.^7^ | 2019 | 2017 | Bahrain | Asia | All ages | 829 | Disc diffusion | 300 | 43 | 5.2 |
| Altamimi et al.^8^ | 2023 | 2019-2020 | Saudi Arabia | Asia | Ages 0 to 15 | 599 | Disc diffusion | 300 | 18 | 3.2 |
| Anvari et al.^9^ | 2014 | 2008-2010 | Iran | Asia | All ages | 514 | Disc diffusion | 100 | 26 | 5.3 |
| Araújo et al.^10^ | 2011 | 2007-2009 | Brazil | South America | Females aged >=14 | 415 | Disc diffusion | Not stated | 39 | 9.4 |
| Arredondo-García and Amábile-Cuevas^11^ | 2008 | Not stated | Mexico | North America | Pregnant females | 907 | Disc diffusion | 300 | 67 | 7.4 |
| Bakhtiari et al.^12^ | 2020 | Not stated | Iran | Asia | All ages | 113 | Disc diffusion | 300 | 4 | 3.5 |
| Biset et al.^14^ | 2020 | 2017 | Ethiopia | Africa | Pregnant females | 30 | Disc diffusion | 300 | 0 | 0.0 |
| Campbell et al.^15^ | 2022 | 2017-2021 | Sierra Leone | Africa | All ages | 36 | Disc diffusion | Not stated | 10 | 28.0 |
| Cunha et al.^18^ | 2016 | 2007-2010 | Brazil | South America | All ages | 653 | Disc diffusion | Not stated | 43 | 6.6 |
| Curtis et al.^19^ | 2023 | 2019-2021 | Australia | Oceania | Ages 47 to 76 | 48 | Disc diffusion | Not stated | 3 | 6.7 |
| Dadi et al.^20^ | 2018 | Not stated | Ethiopia | Africa | All ages | 200 | Disc diffusion | 300 | 10 | 5.0 |
| Daoud et al.^21^ | 2020 | 2012-2018 | Tunisia | Africa | All ages | 1146 | Disc diffusion | 100 | 28 | 2.4 |
| De Francesco et al.^22^ | 2007 | 2002-2005 | Italy | Europe | Unknown | 3720 | VITEK-2 | Not stated | 290 | 7.8 |
| Dehbanipour et al.^23^ | 2016 | 2012-2013 | Iran | Asia | All ages | 135 | Disc diffusion | 300 | 19 | 14.1 |
| Demilie et al.^24^ | 2012 | 2010-2011 | Ethiopia | Africa | Pregnant females | 16 | Disc diffusion | 300 | 1 | 6.3 |
| Derebe et al.^25^ | 2025 | 2020-2022 | Ethiopia | Africa | Pregnant females | 12 | VITEK-2 | Not stated | 0 | 0.0 |
| Farajnia et al.^26^ | 2009 | Not stated | Iran | Asia | Ages 18 months to 65 years | 504 | Disc diffusion | Not stated | 65 | 12.9 |
| Farshad et al.^27^ | 2012 | Not stated | Iran | Asia | Ages 1 month to 14 years | 96 | Disc diffusion | 300 | 3 | 3.2 |
| Fasugba et al.^28^ | 2016 | 2009-2013 | Australia | Oceania | All ages | 1599 | VITEK-2 | 300 | 43 | 2.7 |
| Gales et al.^29^ | 2002 | 1997-2000 | Multiple | North America + South America | All ages | 1221 | Microdilution | 32/64 | 123 | 10.1 |
| Gangcuangco et al.^30^ | 2015 | 2010-2011 | Phillippines | Asia | Females aged >=18 | 179 | VITEK-2 | Not stated | 9 | 5.1 |
| Haindongo et al.^33^ | 2022 | 2016-2017 | Namibia | Africa | Females, all ages | 5568 | VITEK-2 | 300 | 607 | 10.9 |
| Haque et al.^34^ | 2015 | 2012 | Bangladesh | Asia | All ages | 118 | Disc diffusion | 300 | 19 | 16.1 |
| Hasan et al.^35^ | 2023 | 2019-2020 | India | Asia | All ages | 736 | VITEK-2 | 50/90 | 44 | 6.0 |
| Ho et al.^36^ | 2010 | 2006-2008 | Hong Kong | Asia | Females aged >=18 | 271 | Disc diffusion | Not stated | 18 | 6.6 |
| Ilić et al.^37^ | 2011 | 2000-2007 | Croatia | Europe | Children | 917 | Disc diffusion | Not stated | 4 | 0.4 |
| Iqbal et al.^38^ | 2021 | 2018-2019 | Pakistan | Asia | Ages 0 to 13 | 63 | Disc diffusion | Not stated | 13 | 21.3 |
| Issazadeh et al.^39^ | 2015 | 2012-2013 | Iran | Asia | Ages 1 to 85 | 110 | Disc diffusion | Not stated | 48 | 43.6 |
| Jadhav et al.^40^ | 2011 | 2009-2010 | India | Asia | All ages | 150 | Disc diffusion | 300 | 64 | 42.7 |
| Jalilian et al.^41^ | 2014 | 2011-2012 | Iran | Asia | All ages | 801 | Disc diffusion | 300 | 112 | 14.0 |
| Karlowsky et al.^42^ | 2001 | 1999 | United States of America | North America | Females, all ages | 3505 | Microdilution | Not stated | 18 | 0.5 |
| Kebede et al.^43^ | 2025 | 2024 | Ethiopia | Africa | All ages | 21 | Disc diffusion | 300 | 2 | 9.5 |
| Khan et al.^44^ | 2023 | 2018-2019 | Pakistan | Asia | All ages | 23 | Disc diffusion | 300 | 5 | 21.8 |
| Kothari and Sagar^45^ | 2008 | 2005 | India | Asia | Non-pregnant females, ages 18 to 72 | 361 | Disc diffusion | Not stated | 88 | 24.4 |
| Maraki et al.^48^ | 2013 | 2005-2010 | Greece | Europe | Ages >14 | 2762 | Disc diffusion + VITEK-2 | Not stated | 185 | 6.7 |
| Matute et al.^49^ | 2004 | 2002 | Nicaragua | North America | All sexes and ages | 34 | Disc diffusion | Not stated | 0 | 0.0 |
| Mohamed et al.^50^ | 2023 | 2022 | Somalia | Africa | Pregnant females | 42 | Disc diffusion | Not stated | 6 | 14.3 |
| Mohamed et al.^51^ | 2020 | 2019 | Somalia | Africa | All ages | 34 | Disc diffusion | Not stated | 5 | 14.7 |
| Mohapatra et al.^52^ | 2023 | Not stated | India | Asia | Ages 18 months to 18 years | 54 | VITEK-2 | 32/128 | 6 | 11.2 |
| Mouanga Ndzime et al.^54^ | 2021 | 2018-2019 | Gabon | Africa | All ages | 78 | Disc diffusion | Not stated | 1 | 1.3 |
| Munkhdelger et al.^55^ | 2017 | 2012-2013 | Mongolia | Asia | Not stated | 148 | Disc diffusion | 300 | 8 | 5.4 |
| Mwaka et al.^56^ | 2011 | Not stated | Uganda | Africa | Non-pregnant females, ages >=18 | 28 | Disc diffusion | 300 | 0 | 0.0 |
| Nakandi et al.^57^ | 2025 | 2019-2023 | Uganda | Africa | All ages | 188 | Disc diffusion | 300 | 32 | 17.1 |
| Neuzillet et al.^58^ | 2012 | 2003-2006 | France | Europe | Females, all ages | 402 | Disc diffusion | Not stated | 4 | 1.0 |
| Norouzian et al.^60^ | 2019 | 2016-2017 | Iran | Asia | All ages | 261 | Disc diffusion | 30 | 0 | 0.0 |
| Odongo et al.^61^ | 2020 | 2018 | Uganda | Africa | All ages | 10 | Disc diffusion | 300 | 3 | 30.0 |
| Olorunmola et al.^62^ | 2013 | 2003-2005 | Nigeria | Africa | All ages | 137 | Disc diffusion | 300 | 10 | 7.3 |
| Ong et al.^63^ | 2021 | 2014-2019 | United Kingdom | Europe | All ages | 712004 | Semi-automated urine culture | Not stated | 8544 | 1.2 |
| Orrett^64^ | 2003 | 1996-1999 | Trinidad | Caribbean | All ages | 511 | Disc diffusion | 300 | 80 | 15.6 |
| Prakash and Saxena^65^ | 2013 | 2011-2013 | India | Asia | Ages 15 to >48 | 66 | Disc diffusion | Not stated | 17 | 25.8 |
| Rafalskiy et al.^66^ | 2020 | 2017 | Russia | Europe + Asia | All ages | 20303 | VITEK-2 | Not stated | 914 | 4.5 |
| Raza et al.^67^ | 2011 | 2009-2010 | Nepal | Asia | All ages | 515 | Disc diffusion | 30 | 45 | 8.7 |
| Rezaee and Abdinia^68^ | 2015 | 2010-2014 | Iran | Asia | Children | 671 | Disc diffusion | 300 | 74 | 11.1 |
| Rizvi et al.^69^ | 2024 | 2022 | India | Asia | Not stated | 7587 | Disc diffusion | Not stated | 1017 | 13.4 |
| Sedighi et al.^70^ | 2015 | 2010-2011 | Iran | Asia | Children | 120 | Disc diffusion | 300 | 0 | 0.0 |
| Shaifali et al.^71^ | 2012 | Not stated | India | Asia | Females, ages >=15 | 46 | Disc diffusion | Not stated | 6 | 13.1 |
| Sundvall et al.^73^ | 2014 | 2003-2012 | Sweden | Europe | Nursing home residents | 260 | Disc diffusion | Not stated | 2 | 0.8 |
| Tutone et al.^74^ | 2022 | 2019 | Multiple | Europe | Not stated | 2060 | Disc diffusion | 100 | 31 | 1.5 |
| Vakilzadeh et al.^75^ | 2020 | 2017-2019 | Iran | Asia | All ages | 121 | Disc diffusion | Not stated | 13 | 10.7 |
| Watts et al.^76^ | 2020 | 2017-2018 | United Kingdom | Europe | Ages >=18 | 110 | VITEK-2 | Not stated | 1 | 0.9 |

UPEC = uropathogenic *Escherichia coli*, AST = antibiotic susceptibility testing

**Table S4.** Country-wise pooled prevalence/prevalence of nitrofurantoin-resistant uropathogenic *Escherichia coli* (UPEC) isolates

| **Country** | **Number of Studies** | **Total UPEC Isolates** | **Nitrofurantoin-Resistant UPEC Isolates** | **Pooled Prevalence** | **CI Lower** | **CI Upper** | ***Q-*value** | ***I^2^-*value** |
| --- | --- | --- | --- | --- | --- | --- | --- | --- |
| Australia | 2 | 1647 | 46 | 0.032 | 0 | 0.497 | 2.05 | 51.1 |
| Bahrain | 1 | 829 | 43 | 0.052 |  |  |  |  |
| Bangladesh | 1 | 118 | 19 | 0.161 |  |  |  |  |
| Belgium | 1 | 367 | 5 | 0.014 |  |  |  |  |
| Brazil | 2 | 1068 | 39 | 0.078 | 0 | 0.334 | 2.79 | 64.1 |
| Croatia | 1 | 917 | 4 | 0.004 |  |  |  |  |
| Ethiopia | 5 | 279 | 13 | 0.031 | 0.004 | 0.074 | 4.12 | 3 |
| France | 1 | 402 | 4 | 0.01 |  |  |  |  |
| Gabon | 1 | 78 | 1 | 0.013 |  |  |  |  |
| Greece | 1 | 2762 | 185 | 0.067 |  |  |  |  |
| Hong Kong | 1 | 271 | 18 | 0.066 |  |  |  |  |
| India | 7 | 9000 | 1242 | 0.183 | 0.084 | 0.307 | 151.97 | 96.1 |
| Iran | 11 | 2819 | 364 | 0.082 | 0.025 | 0.166 | 245.31 | 95.9 |
| Italy | 2 | 4045 | 298 | 0.049 | 0 | 0.703 | 17.55 | 94.3 |
| Mexico | 1 | 907 | 67 | 0.074 |  |  |  |  |
| Mongolia | 1 | 148 | 8 | 0.054 |  |  |  |  |
| Namibia | 1 | 5568 | 607 | 0.109 |  |  |  |  |
| Nepal | 1 | 515 | 45 | 0.087 |  |  |  |  |
| Nicaragua | 1 | 34 | 0 | 0 |  |  |  |  |
| Nigeria | 2 | 287 | 43 | 0.139 | 0 | 1 | 12.8 | 92.2 |
| Pakistan | 3 | 166 | 21 | 0.133 | 0 | 0.51 | 12.6 | 84.1 |
| Philippines | 1 | 179 | 9 | 0.05 |  |  |  |  |
| Russia | 2 | 20998 | 927 | 0.031 | 0 | 0.365 | 14.79 | 93.2 |
| Saudi Arabia | 2 | 700 | 21 | 0.029 | 0.016 | 0.045 | 0.03 | 0 |
| Sierra Leone | 1 | 36 | 10 | 0.278 |  |  |  |  |
| Somalia | 2 | 76 | 11 | 0.145 | 0.108 | 0.185 | 0.01 | 0 |
| Spain | 1 | 435 | 0 | 0 |  |  |  |  |
| Sweden | 1 | 260 | 2 | 0.008 |  |  |  |  |
| Trinidad | 1 | 511 | 80 | 0.157 |  |  |  |  |
| Tunisia | 1 | 1146 | 28 | 0.024 |  |  |  |  |
| Uganda | 3 | 226 | 35 | 0.107 | 0 | 0.697 | 12.91 | 84.5 |
| United Kingdom | 3 | 712354 | 8550 | 0.011 | 0.005 | 0.019 | 1.69 | 0 |
| United States | 1 | 3505 | 18 | 0.005 |  |  |  |  |

CI = confidence interval

**Table S5.**  Sensitivity analysis of the included studies by stepwise study omission

| **Study Removed** | **Pooled Prevalence** | **CI Lower** | **CI Upper** | ***Q*-value** | ***p*-value** | ***I^2^*-value** |
| --- | --- | --- | --- | --- | --- | --- |
| Abdu et al. 2018^2^ | 0.067 | 0.047 | 0.095 | 13094.332 | 0.000 | 100% |
| Alanazi et al. 2018^5^ | 0.069 | 0.049 | 0.098 | 13256.882 | 0.000 | 100% |
| Ali et al. 2014^6^ | 0.069 | 0.049 | 0.098 | 13256.025 | 0.000 | 100% |
| Al Khawaja et al. 2019^7^ | 0.069 | 0.048 | 0.098 | 13226.948 | 0.000 | 100% |
| Altamimi et al. 2023^8^ | 0.07 | 0.049 | 0.098 | 13255.786 | 0.000 | 100% |
| Anvari et al. 2014^9^ | 0.069 | 0.048 | 0.098 | 13239.709 | 0.000 | 100% |
| Araújo et al. 2011^10^ | 0.068 | 0.048 | 0.096 | 13176.878 | 0.000 | 100% |
| Arredondo-García and Amábile-Cuevas 2008^11^ | 0.069 | 0.048 | 0.097 | 13162.149 | 0.000 | 100% |
| Bakhtiari et al. 2020^12^ | 0.069 | 0.049 | 0.098 | 13256.102 | 0.000 | 100% |
| Biset et al. 2020^14^ | 0.07 | 0.049 | 0.098 | 13257.406 | 0.000 | 100% |
| Campbell et al. 2022^15^ | 0.067 | 0.047 | 0.094 | 13197.283 | 0.000 | 100% |
| Cunha et al. 2016^18^ | 0.069 | 0.048 | 0.097 | 13207.058 | 0.000 | 100% |
| Curtis et al. 2022^19^ | 0.069 | 0.048 | 0.097 | 13252.873 | 0.000 | 100% |
| Dadi et al. 2018^20^ | 0.069 | 0.048 | 0.097 | 13250.177 | 0.000 | 100% |
| Daoud et al. 2020^21^ | 0.07 | 0.049 | 0.099 | 13257.271 | 0.000 | 100% |
| De Francesco et al. 2007^22^ | 0.068 | 0.048 | 0.097 | 12805.444 | 0.000 | 100% |
| Dehbanipour et al. 2016^23^ | 0.068 | 0.048 | 0.096 | 13192.791 | 0.000 | 100% |
| Demilie et al. 2012^24^ | 0.069 | 0.048 | 0.097 | 13254.716 | 0.000 | 100% |
| Derebe et al. 2025^25^ | 0.069 | 0.049 | 0.097 | 13257.331 | 0.000 | 100% |
| Farajnia et al. 2009^26^ | 0.068 | 0.048 | 0.096 | 13061.406 | 0.000 | 100% |
| Farshad et al. 2012^27^ | 0.069 | 0.049 | 0.098 | 13256.727 | 0.000 | 100% |
| Fasugba et al. 2016^28^ | 0.07 | 0.049 | 0.099 | 13256.146 | 0.000 | 100% |
| Gales et al. 2002^29^ | 0.068 | 0.048 | 0.096 | 12981.898 | 0.000 | 100% |
| Gangcuangco et al. 2015^30^ | 0.069 | 0.048 | 0.097 | 13250.708 | 0.000 | 100% |
| Haindongo et al. 2022^33^ | 0.068 | 0.048 | 0.096 | 11709.578 | 0.000 | 100% |
| Haque et al. 2015^34^ | 0.068 | 0.048 | 0.095 | 13183.805 | 0.000 | 100% |
| Hasan et al. 2023^35^ | 0.069 | 0.048 | 0.097 | 13214.75 | 0.000 | 100% |
| Ho et al. 2010^36^ | 0.069 | 0.048 | 0.097 | 13235.176 | 0.000 | 100% |
| Ilić et al. 2011^37^ | 0.072 | 0.05 | 0.101 | 13246.576 | 0.000 | 100% |
| Iqbal et al. 2021^38^ | 0.067 | 0.047 | 0.095 | 13194.275 | 0.000 | 100% |
| Issazadeh et al. 2015^39^ | 0.066 | 0.047 | 0.093 | 12922.146 | 0.000 | 100% |
| Jadhav et al. 2011^40^ | 0.066 | 0.047 | 0.093 | 12813.707 | 0.000 | 100% |
| Jalilian et al. 2014^41^ | 0.068 | 0.048 | 0.096 | 12890.262 | 0.000 | 100% |
| Karlowsky et al. 2001^42^ | 0.072 | 0.05 | 0.101 | 13216.811 | 0.000 | 100% |
| Kebede et al. 2025^43^ | 0.068 | 0.048 | 0.096 | 13251.065 | 0.000 | 100% |
| Khan et al. 2023^44^ | 0.067 | 0.047 | 0.095 | 13230.088 | 0.000 | 100% |
| Kothari and Sagar, 2008^45^ | 0.067 | 0.047 | 0.095 | 12794.027 | 0.000 | 100% |
| Maraki et al. 2013^48^ | 0.069 | 0.048 | 0.097 | 13036.394 | 0.000 | 100% |
| Matute et al. 2004^49^ | 0.07 | 0.049 | 0.098 | 13257.355 | 0.000 | 100% |
| Mohamed et al. 2023^50^ | 0.068 | 0.048 | 0.096 | 13234.758 | 0.000 | 100% |
| Mohamed et al. 2020^51^ | 0.068 | 0.048 | 0.096 | 13237.516 | 0.000 | 100% |
| Mohapatra et al. 2023^52^ | 0.068 | 0.048 | 0.096 | 13240.024 | 0.000 | 100% |
| Mouanga Ndzime et al. 2021^54^ | 0.07 | 0.049 | 0.099 | 13257.415 | 0.000 | 100% |
| Munkhdelger et al. 2017^55^ | 0.069 | 0.048 | 0.097 | 13250.249 | 0.000 | 100% |
| Mwaka et al. 2011^56^ | 0.07 | 0.049 | 0.098 | 13257.427 | 0.000 | 100% |
| Nakandi et al. 2025^57^ | 0.068 | 0.047 | 0.095 | 13129.053 | 0.000 | 100% |
| Neuzillet et al. 2012^58^ | 0.071 | 0.05 | 0.1 | 13255.098 | 0.000 | 100% |
| Norouzian et al. 2019^60^ | 0.071 | 0.05 | 0.1 | 13254.335 | 0.000 | 100% |
| Odongo et al. 2020^61^ | 0.067 | 0.047 | 0.095 | 13236.105 | 0.000 | 100% |
| Olorunmola et al. 2013^62^ | 0.069 | 0.048 | 0.097 | 13242.125 | 0.000 | 100% |
| Ong et al. 2021^63^ | 0.077 | 0.064 | 0.093 | 1815.403 | 0.000 | 100% |
| Orrett 2003^64^ | 0.068 | 0.048 | 0.095 | 12963.788 | 0.000 | 100% |
| Prakash and Saxena 2013^65^ | 0.067 | 0.047 | 0.095 | 13162.128 | 0.000 | 100% |
| Rafalskiy et al. 2020^66^ | 0.069 | 0.047 | 0.1 | 12797.379 | 0.000 | 100% |
| Raza et al. 2011^67^ | 0.068 | 0.048 | 0.097 | 13173.791 | 0.000 | 100% |
| Rezaee and Abdinia 2015^68^ | 0.068 | 0.048 | 0.096 | 13071.307 | 0.000 | 100% |
| Rizvi et al. 2024^69^ | 0.068 | 0.048 | 0.095 | 9874.047 | 0.000 | 100% |
| Sedighi et al. 2015^70^ | 0.07 | 0.05 | 0.099 | 13255.976 | 0.000 | 100% |
| Shaifali et al. 2012^71^ | 0.068 | 0.048 | 0.096 | 13236.717 | 0.000 | 100% |
| Sundvall et al. 2014^73^ | 0.071 | 0.05 | 0.1 | 13255.53 | 0.000 | 100% |
| Tutone et al. 2022^74^ | 0.07 | 0.049 | 0.099 | 13252.198 | 0.000 | 100% |
| Vakilzadeh et al. 2020^75^ | 0.068 | 0.048 | 0.096 | 13223.959 | 0.000 | 100% |
| Watts et al. 2020^76^ | 0.07 | 0.049 | 0.099 | 13257.042 | 0.000 | 100% |

CI = confidence interval

**References**

1. Munn Z, Moola S, Lisy K, Riitano D, Tufanaru C. Chapter 5: Systematic reviews of prevalence and incidence. In: *JBI Manual for Evidence Synthesis*. JBI, 2020. Available at: https://jbi-global-wiki.refined.site/space/MANUAL/355863557/Previous+versions?attachment=/download/attachments/355863557/JBI_Reviewers_Manual_2020June.pdf&type=application/pdf&filename=JBI_Reviewers_Manual_2020June.pdf#page=175. Accessed June 2, 2025.

2. Abdu A, Kachallah M, Bolus DY. Antibiotic susceptibility patterns of Uropathogenic Escherichia coli among patients with urinary tract infections in a tertiary care hospital in Maiduguri, North Eastern, Nigeria. *J Biosci Biotechnol Discov* 2018; **3**: 14–24.

3. Abduzaimovic A, Aljicevic M, Rebic V, Vranic SM, Abduzaimovic K, Sestic S. Antibiotic Resistance in Urinary Isolates of Escherichia coli. *Mater Sociomed* 2016; **28**: 416–9.

4. Adib N, Ghanbarpour R, Solatzadeh H, Alizade H. Antibiotic resistance profile and virulence genes of uropathogenic Escherichia coli isolates in relation to phylogeny. *Trop Biomed* 2014; **31**: 17–25.

5. Alanazi MQ, Alqahtani FY, Aleanizy FS. An evaluation of E. coli in urinary tract infection in emergency department at KAMC in Riyadh, Saudi Arabia: retrospective study. *Ann Clin Microbiol Antimicrob* 2018; **17**: 3.

6. Ali I, Kumar N, Ahmed S, Dasti JI. Antibiotic resistance in uropathogenic e. Coli strains isolated from non-hospitalized patients in Pakistan. *J Clin Diagn Res* 2014; **8**: DC01-04.

7. Al Khawaja S, Al Aagha R, Saeed NK, Fawzy N. Prevalence of Resistant Uropathogenic Escherichia coli in Bahrain: A Community Based Study. *J Bah Med Soc* 2019. Available at: http://www.bhmedsoc.com/jbms/view-article.php?Article_Unique_Id=JBMS146. Accessed June 2, 2025.

8. Altamimi I, Almazyed A, Alshammary S, *et al.* Bacterial Pathogens and Antimicrobial Susceptibility Patterns of Urinary Tract Infections in Children during COVID-19 2019-2020: A Large Tertiary Care Center in Saudi Arabia. *Children (Basel)* 2023; **10**: 971.

9. Anvari MS, Naderan M, Boroumand MA, Shoar S, Bakhshi R, Naderan M. Microbiologic Spectrum and Antibiotic Susceptibility Pattern among Patients with Urinary and Respiratory Tract Infection. *Int J Microbiol* 2014; **2014**: 682304.

10. Araújo SMHA, Mourão TC, Oliveira JL, *et al.* Antimicrobial resistance of uropathogens in women with acute uncomplicated cystitis from primary care settings. *Int Urol Nephrol* 2011; **43**: 461–6.

11. Arredondo-García JL, Amábile-Cuevas CF. High resistance prevalence towards ampicillin, co-trimoxazole and ciprofloxacin, among uropathogenic Escherichia coli isolates in Mexico City. *J Infect Dev Ctries* 2008; **2**: 350–3.

12. Bakhtiari S, Mahmoudi H, Seftjani SK, *et al.* Antibiotic resistance pattern and phylogenetic groups of the uropathogenic Escherichia coli isolates from urinary tract infections in Hamedan, west of Iran. *Iran J Microbiol* 2020; **12**: 388–94.

13. Bhola P, Mvelase NR, Balakrishna Y, Mlisana KP, Swe Swe-Han K. Antimicrobial susceptibility patterns of uropathogens isolated from pregnant women in KwaZulu-Natal Province: 2011 - 2016. *S Afr Med J* 2020; **110**: 872–6.

14. Biset S, Moges F, Endalamaw D, Eshetie S. Multi-drug resistant and extended-spectrum β-lactamases producing bacterial uropathogens among pregnant women in Northwest Ethiopia. *Ann Clin Microbiol Antimicrob* 2020; **19**: 25.

15. Campbell JSO, van Henten S, Koroma Z, *et al.* Culture Requests and Multi-Drug Resistance among Suspected Urinary Tract Infections in Two Tertiary Hospitals in Freetown, Sierra Leone (2017-21): A Cross-Sectional Study. *Int J Environ Res Public Health* 2022; **19**: 4865.

16. Caracciolo A, Bettinelli A, Bonato C, *et al.* Antimicrobial resistance among Escherichia coli that cause childhood community-acquired urinary tract infections in Northern Italy. *Ital J Pediatr* 2011; **37**: 3.

17. Carmona-Cartaya Y, Hidalgo-Benito M, Borges-Mateus LM, Pereda-Novales N, González-Molina MK, Quiñones-Pérez D. Community-Acquired Uropathogenic Escherichia coli, Antimicrobial Susceptibility, and Extended-Spectrum Beta-Lactamase Detection. *MEDICC Rev* 2022; **24**: 20–5.

18. Cunha MA, Assunção GLM, Medeiros IM, Freitas MR. Antibiotic resistance patterns of urinary tract infections in a Northeastern Brazilian capital. *Rev Inst Med Trop Sao Paulo* 2016; **58**: 2.

19. Curtis SJ, Kwong JC, Chaung YL, *et al.* Resistance to first-line antibiotic therapy among patients with uncomplicated acute cystitis in Melbourne, Australia: prevalence, predictors and clinical impact. *JAC Antimicrob Resist* 2024; **6**: dlad145.

20. Dadi BR, Abebe T, Zhang L, Mihret A, Abebe W, Amogne W. Drug resistance and plasmid profile of uropathogenic Escherichia coli among urinary tract infection patients in Addis Abeba. *J Infect Dev Ctries* 2018; **12**: 608–15.

21. Daoud N, Hamdoun M, Hannachi H, Gharsallah C, Mallekh W, Bahri O. Antimicrobial Susceptibility Patterns of Escherichia coli among Tunisian Outpatients with Community-Acquired Urinary Tract Infection (2012-2018). *Curr Urol* 2020; **14**: 200–5.

22. De Francesco MA, Ravizzola G, Peroni L, Negrini R, Manca N. Urinary tract infections in Brescia, Italy: etiology of uropathogens and antimicrobial resistance of common uropathogens. *Med Sci Monit* 2007; **13**: BR136-144.

23. Dehbanipour R, Rastaghi S, Sedighi M, Maleki N, Faghri J. High prevalence of multidrug-resistance uropathogenic Escherichia coli strains, Isfahan, Iran. *J Nat Sci Biol Med* 2016; **7**: 22–6.

24. Demilie T, Beyene G, Melaku S, Tsegaye W. Urinary bacterial profile and antibiotic susceptibility pattern among pregnant women in north west ethiopia. *Ethiop J Health Sci* 2012; **22**: 121–8.

25. Derebe MM, Paladhi UR, Workneh F, *et al.* Urinary tract infections among pregnant women in rural West Amhara, Ethiopia: Prevalence, bacterial etiology, risk factors, and antimicrobial resistance patterns. *Res Sq* 2025: rs.3.rs-5737078.

26. Farajnia S, Alikhani MY, Ghotaslou R, Naghili B, Nakhlband A. Causative agents and antimicrobial susceptibilities of urinary tract infections in the northwest of Iran. *Int J Infect Dis* 2009; **13**: 140–4.

27. Farshad S, Ranjbar R, Japoni A, Hosseini M, Anvarinejad M, Mohammadzadegan R. Microbial susceptibility, virulence factors, and plasmid profiles of uropathogenic Escherichia coli strains isolated from children in Jahrom, Iran. *Arch Iran Med* 2012; **15**: 312–6.

28. Fasugba O, Mitchell BG, Mnatzaganian G, Das A, Collignon P, Gardner A. Five-Year Antimicrobial Resistance Patterns of Urinary Escherichia coli at an Australian Tertiary Hospital: Time Series Analyses of Prevalence Data. *PLoS One* 2016; **11**: e0164306.

29. Gales AC, Sader HS, Jones RN, SENTRY Participants Group (Latin America). Urinary tract infection trends in Latin American hospitals: report from the SENTRY antimicrobial surveillance program (1997-2000). *Diagn Microbiol Infect Dis* 2002; **44**: 289–99.

30. Gangcuangco LM, Alejandria M, Henson KE, *et al.* Prevalence and risk factors for trimethoprim-sulfamethoxazole-resistant Escherichia coli among women with acute uncomplicated urinary tract infection in a developing country. *Int J Infect Dis* 2015; **34**: 55–60.

31. Gatya Al-Mayahie SM, Al-Guranie DRT, Hussein AA, Bachai ZA. Prevalence of common carbapenemase genes and multidrug resistance among uropathogenic Escherichia coli phylogroup B2 isolates from outpatients in Wasit Province/ Iraq. *PLoS One* 2022; **17**: e0262984.

32. Haghighatpanah M, Mojtahedi A. Characterization of antibiotic resistance and virulence factors of Escherichia coli strains isolated from Iranian inpatients with urinary tract infections. *Infect Drug Resist* 2019; **12**: 2747–54.

33. Haindongo EH, Funtua B, Singu B, *et al.* Antimicrobial resistance among bacteria isolated from urinary tract infections in females in Namibia, 2016-2017. *Antimicrob Resist Infect Control* 2022; **11**: 33.

34. Haque R, Akter MostL, Salam MdA. Prevalence and susceptibility of uropathogens: a recent report from a teaching hospital in Bangladesh. *BMC Res Notes* 2015; **8**: 416.

35. Hasan AS, Garg R, Nasimuddin S, *et al.* Rising Resistance In Uropathogens With An Indication Of Nitrofurantoin Mic Creep. *J Ayub Med Coll Abbottabad* 2023; **35**: 54–9.

36. Ho P, Yip K, Chow K, Lo JYC, Que T, Yuen K. Antimicrobial resistance among uropathogens that cause acute uncomplicated cystitis in women in Hong Kong: a prospective multicenter study in 2006 to 2008. *Diagn Microbiol Infect Dis* 2010; **66**: 87–93.

37. Ilić T, Gračan S, Arapović A, Capkun V, Subat-Dežulović M, Saraga M. Changes in bacterial resistance patterns in children with urinary tract infections on antimicrobial prophylaxis at University Hospital in Split. *Med Sci Monit* 2011; **17**: CR355-361.

38. Iqbal Z, Mumtaz MZ, Malik A. Extensive drug-resistance in strains of Escherichia coli and Klebsiella pneumoniae isolated from paediatric urinary tract infections. *J Taibah Univ Med Sci* 2021; **16**: 565–74.

39. Issazadeh K, Naghibi SN, Khoshkholgh-Pahlaviani MRM. Drug Resistance and Serotyping of Uropathogenic Escherichia coli Among Patients With Urinary Tract Infection in Rasht, Iran. *Zahedan J Res Med Sci* 2015; **17**. Available at: https://brieflands.com/articles/zjrms-989.html. Accessed June 2, 2025.

40. Jadhav S, Hussain A, Devi S, *et al.* Virulence characteristics and genetic affinities of multiple drug resistant uropathogenic Escherichia coli from a semi urban locality in India. *PLoS One* 2011; **6**: e18063.

41. Jalilian S, Farahani A, Mohajeri P. Antibiotic resistance in uropathogenic Escherichia coli isolated from urinary tract infections out-patients in Kermanshah. *Int J Med Public Health* 2014; **4**: 75.

42. Karlowsky JA, Jones ME, Thornsberry C, Critchley I, Kelly LJ, Sahm DF. Prevalence of antimicrobial resistance among urinary tract pathogens isolated from female outpatients across the US in 1999. *Int J Antimicrob Agents* 2001; **18**: 121–7.

43. Kebede D, Shiferaw Y, Kebede E, Demsiss W. Antimicrobial susceptibility and risk factors of uropathogens in symptomatic urinary tract infection cases at Dessie Referral Hospital, Ethiopia. *BMC Microbiol* 2025; **25**: 126.

44. Khan MA, Rahman AU, Khan B, *et al.* Antibiotic Resistance Profiling and Phylogenicity of Uropathogenic Bacteria Isolated from Patients with Urinary Tract Infections. *Antibiotics (Basel)* 2023; **12**: 1508.

45. Kothari A, Sagar V. Antibiotic resistance in pathogens causing community-acquired urinary tract infections in India: a multicenter study. *J Infect Dev Ctries* 2008; **2**: 354–8.

46. Kumar M N, Bhat S, Bhat K A, Saralaya V, Shenoy Mulki S. Characterization of virulence factors and antibiotic resistance pattern of uropathogenic Escherichia coli strains in a tertiary care center. *F1000Res* 2022; **11**: 1163.

47. Malik S, Rana JS, Nehra K. Prevalence and Antibiotic Susceptibility Pattern of Uropathogenic Escherichia Coli Strains in Sonipat Region of Haryana in India. *Biomedical and Biotechnology Research Journal (BBRJ)* 2021; **5**: 80–7.

48. Maraki S, Mantadakis E, Michailidis L, Samonis G. Changing antibiotic susceptibilities of community-acquired uropathogens in Greece, 2005-2010. *J Microbiol Immunol Infect* 2013; **46**: 202–9.

49. Matute AJ, Hak E, Schurink C a. M, *et al.* Resistance of uropathogens in symptomatic urinary tract infections in León, Nicaragua. *Int J Antimicrob Agents* 2004; **23**: 506–9.

50. Mohamed FY, Dahie HA, Mohamoud JH, Adam MH, Dirie HM. Prevalence, antimicrobial susceptibility profile, and associated risk factors of uropathogenic Escherichia coli among pregnant women attending Dr. Sumait Hospital Mogadishu, Somalia. *Front Public Health* 2023; **11**: 1203913.

51. Mohamed MA, Abdifetah O, Hussein FA, Karie SA. Antibiotic resistance pattern of Escherichia coli isolates from outpatients with urinary tract infections in Somalia. *J Infect Dev Ctries* 2020; **14**: 284–9.

52. Mohapatra S, Ghosh D, Vivekanandan P, *et al.* Genome profiling of uropathogenic E. coli from strictly defined community-acquired UTI in paediatric patients: a multicentric study. *Antimicrob Resist Infect Control* 2023; **12**: 36.

53. Momtaz H, Karimian A, Madani M, *et al.* Uropathogenic Escherichia coli in Iran: serogroup distributions, virulence factors and antimicrobial resistance properties. *Ann Clin Microbiol Antimicrob* 2013; **12**: 8.

54. Mouanga Ndzime Y, Onanga R, Kassa Kassa RF, *et al.* Epidemiology of Community Origin Escherichia coli and Klebsiella pneumoniae Uropathogenic Strains Resistant to Antibiotics in Franceville, Gabon. *Infect Drug Resist* 2021; **14**: 585–94.

55. Munkhdelger Y, Gunregjav N, Dorjpurev A, Juniichiro N, Sarantuya J. Detection of virulence genes, phylogenetic group and antibiotic resistance of uropathogenic Escherichia coli in Mongolia. *J Infect Dev Ctries* 2017; **11**: 51–7.

56. Mwaka A, Mayanja-Kizza H, Kigonya E, Kaddu-Mulindwa D. Bacteriuria among adult non-pregnant women attending Mulago hospital assessment centre in Uganda. *Afr Health Sci* 2011; **11**: 182–9.

57. Nakandi RM, Kakeeto P, Kihumuro RB, *et al.* Antibiotic susceptibility patterns of bacterial uropathogens at a private tertiary hospital in Uganda: a retrospective study. *BMC Infect Dis* 2025; **25**: 605.

58. Neuzillet Y, Naber KG, Schito G, Gualco L, Botto H. French results of the ARESC study: clinical aspects and epidemiology of antimicrobial resistance in female patients with cystitis. Implications for empiric therapy. *Med Mal Infect* 2012; **42**: 66–75.

59. RM N, Mariappan S, Sekar U, K V L A. Detection of Virulence Determinants of Uropathogenic Escherichia coli. *Cureus* 2025; **17**: e79116.

60. Norouzian H, Katouli M, Shahrokhi N, Sabeti S, Pooya M, Bouzari S. The relationship between phylogenetic groups and antibiotic susceptibility patterns of Escherichia coli strains isolated from feces and urine of patients with acute or recurrent urinary tract infection. *Iran J Microbiol* 2019; **11**: 478–87.

61. Odongo I, Ssemambo R, Kungu JM. Prevalence of Escherichia Coli and Its Antimicrobial Susceptibility Profiles among Patients with UTI at Mulago Hospital, Kampala, Uganda. *Interdiscip Perspect Infect Dis* 2020; **2020**: 8042540.

62. Olorunmola FO, Kolawole DO, Lamikanra A. Antibiotic resistance and virulence properties in Escherichia coli strains from cases of urinary tract infections. *Afr J Infect Dis* 2013; **7**: 1–7.

63. Ong A, Mahobia N, Browning D, Schembri M, Somani BK. Trends in antibiotic resistance for over 700,000 Escherichia coli positive urinary tract infections over six years (2014–2019) from a university teaching hospital. *Cent European J Urol* 2021; **74**: 249–54.

64. Orrett FA. Antimicrobial susceptibility patterns of urinary pathogens in Trinidad, 1996-1999. *J Natl Med Assoc* 2003; **95**: 352–62.

65. Prakash D, Saxena RS. Distribution and antimicrobial susceptibility pattern of bacterial pathogens causing urinary tract infection in urban community of meerut city, India. *ISRN Microbiol* 2013; **2013**: 749629.

66. Rafalskiy V, Pushkar D, Yakovlev S, *et al.* Distribution and antibiotic resistance profile of key Gram-negative bacteria that cause community-onset urinary tract infections in the Russian Federation: RESOURCE multicentre surveillance 2017 study. *J Glob Antimicrob Resist* 2020; **21**: 188–94.

67. Raza S, Pandey S, Bhatt CP. Microbiological analysis of isolates in Kathmandu Medical College Teaching Hospital, Kathmandu, Nepal. *Kathmandu Univ Med J (KUMJ)* 2011; **9**: 295–7.

68. Rezaee MA, Abdinia B. Etiology and Antimicrobial Susceptibility Pattern of Pathogenic Bacteria in Children Subjected to UTI. *Medicine (Baltimore)* 2015; **94**: e1606.

69. Rizvi M, Malhotra S, Agarwal J, *et al.* Regional variations in antimicrobial susceptibility of community-acquired uropathogenic Escherichia coli in India: Findings of a multicentric study highlighting the importance of local antibiograms. *IJID Reg* 2024; **11**: 100370.

70. Sedighi I, Arabestani MR, Rahimbakhsh A, Karimitabar Z, Alikhani MY. Dissemination of Extended-Spectrum β-Lactamases and Quinolone Resistance Genes Among Clinical Isolates of Uropathogenic Escherichia coli in Children. *Jundishapur J Microbiol* 2015; **8**: e19184.

71. Shaifali I, Gupta U, Mahmood SE, Ahmed J. Antibiotic susceptibility patterns of urinary pathogens in female outpatients. *N Am J Med Sci* 2012; **4**: 163–9.

72. Shehabi AA, Mahafzah AM, Al-Khalili KZ. Antimicrobial resistance and plasmid profiles of urinary Escherichia coli isolates from Jordanian patients. *East Mediterr Health J* 2004; **10**: 322–8.

73. Sundvall P-D, Elm M, Gunnarsson R, *et al.* Antimicrobial resistance in urinary pathogens among Swedish nursing home residents remains low: a cross-sectional study comparing antimicrobial resistance from 2003 to 2012. *BMC Geriatr* 2014; **14**: 30.

74. Tutone M, Bjerklund Johansen TE, Cai T, Mushtaq S, Livermore DM. SUsceptibility and Resistance to Fosfomycin and other antimicrobial agents among pathogens causing lower urinary tract infections: findings of the SURF study. *Int J Antimicrob Agents* 2022; **59**: 106574.

75. Vakilzadeh MM, Heidari A, Mehri A, *et al.* Antimicrobial Resistance among Community-Acquired Uropathogens in Mashhad, Iran. *J Environ Public Health* 2020; **2020**: 3439497.

76. Watts V, Brown B, Ahmed M, *et al.* Routine laboratory surveillance of antimicrobial resistance in community-acquired urinary tract infections adequately informs prescribing policy in England. *JAC Antimicrob Resist* 2020; **2**: dlaa022.

77. Yılmaz N, Ağuş N, Bayram A, *et al.* Antimicrobial susceptibilities of Escherichia coli isolates as agents of community-acquired urinary tract infection (2008-2014). *Turk J Urol* 2016; **42**: 32–6.
